# Revisiting Occupational Safety to Protect Workers Exposed to Antineoplastic Drugs

**DOI:** 10.1177/21650799251391835

**Published:** 2025-11-17

**Authors:** Jaehyun Kweon, Wendie Robbins

**Affiliations:** 1 School of Nursing and Fielding School of Public Health, University of California Los Angeles

**Keywords:** antineoplastic drug exposure, occupational health and safety, healthcare worker, personal protective equipment, workplace safety culture

While antineoplastic drugs (ADs) play a crucial role in cancer treatment, they also pose serious occupational hazards to healthcare personnel exposed during preparation, administration, or indirect contact through contaminated excreta, surfaces, and aerosolized particles (Eisenberg et al., 2024). Although guidelines exist (Srisintorn et al., 2021), safety updates and implementation remain inconsistent, especially among non-nursing staff such as nursing assistants (NAs), pharmacy delivery technicians, environmental services (EVS) workers, dietary staff, and visitors who may unknowingly enter contaminated environments. This paper emphasizes the urgent need for standardized training, improved personal protective equipment (PPE) accessibility, and a workplace safety culture that protects all personnel potentially exposed to hazardous drugs.

Studies have identified gaps in training and PPE adherence among non-RN staff (Walton et al., 2019). NAs often perform high-risk tasks like toileting and linen changes within 48 hours of chemotherapy administration, yet many lack formal instruction on AD-related risks or protective measures. Walton et al. (2019) found NAs seldom used recommended PPE during these tasks. Many were unaware of which patients had received chemotherapy, due to missing or unclear signage, and only about half reported receiving standardized training (Walton et al., 2019).

Environmental exposure remains a critical concern. Hazardous drug residues such as cyclophosphamide and etoposide have been detected on floors, walls, and toilets, even after discharge cleaning, posing exposure risks to anyone entering these spaces (Walton et al., 2025). Flushing uncovered toilets produces aerosol plumes capable of spreading hazardous particles into the air and onto surrounding surfaces, exacerbating exposure risk for staff, patients, and visitors (Eisenberg et al., 2024).

Moreover, behavioral and workplace culture also affect how PPE is used. A study in Thailand found that self-efficacy and a strong workplace safety climate were associated with better PPE adherence, while interpersonal influences and perceived conflict between patient care and personal safety were barriers, particularly for NAs (Srisintorn et al., 2021). These findings highlight that knowledge alone is insufficient. Confidence, clarity of roles, and cultural reinforcement are essential for sustained safety practices.

Healthcare institutions must revisit safety preparedness at the systems level. First, standardized AD safety training should be implemented across disciplines. Occupational health professionals can lead education, ensuring all personnel—including float pool staff, dietary aides, EVS workers, and pharmacy techs—are trained on AD exposure risks, PPE protocols, and safety procedures. Onboarding should emphasize this content, especially for temporary staff assigned to oncology-related units.

Second, PPEs like chemotherapy-rated gloves and gowns must be accessible at the point of care. Visual alerts for recent chemotherapy administration remind staff and visitors to take immediate precautions. Occupational health teams can monitor PPE use and provide guidance. Simple, multilingual information sheets can guide nonclinical personnel and visitors on how to avoid exposure during their time in patient areas.

Third, institutions must cultivate a supportive safety culture. Management and unit champions, and occupational health professionals should reinforce PPE use, model safe practices, and encourage open communication. Safety checklists, daily huddles, and involvement of occupational health and infection control teams in hazardous drug planning integrate precautions into broader safety strategies. These efforts support safety practices and reach all personnel, regardless of role or licensure.

In conclusion, protecting all persons who is exposed to antineoplastic drug in the healthcare setting, including EVS workers, NAs, and visitors requires more than protocol. It requires collaboration, consistency, evaluation, and commitment. By standardizing education, improving PPE access, and embedding safety into workplace culture, healthcare systems can create environments that are safe for all.
